# Retraction: Diabetes and Overexpression of proNGF Cause Retinal Neurodegeneration via Activation of RhoA Pathway

**DOI:** 10.1371/journal.pone.0186748

**Published:** 2017-10-16

**Authors:** The PLOS ONE Editors

After the publication of the article, concerns were raised about figures in the article, as follows:

Figure 2A included a duplicate of the control micrograph shown in Figure 5A in an earlier publication (Mol Vis. 2012;18:2993–3003).

In Figure 4B, the beta-actin band is similar to the actin panel in Figure 4A in the publication in Diabetologia (Diabetologia (2013), 56: 2329–2339).

Figure 4C (proNGF) duplicates an image in Figure 5F in the publication in Diabetologia (Diabetologia (2013), 56: 2329–2339).

Two of these issues were noted in a previous Correction (http://journals.plos.org/plosone/article?id=10.1371/journal.pone.0096960). However, the University of Georgia subsequently investigated this matter in collaboration with Augusta University (formerly Georgia Regents University) and the Charlie Norwood Veterans Affairs Medical Center. The investigation committee found evidence of misconduct, and the University of Georgia’s Office of Research Compliance recommended retraction due to the instances of confirmed image duplication.

In line with the recommendation by the University of Georgia, the *PLOS ONE* editors retract this publication.

## References

[1] Al-Gayyar MMH, Mysona BA, Matragoon S, Abdelsaid MA, El-Azab MF, Shanab AY, et al (2013) Diabetes and Overexpression of proNGF Cause Retinal Neurodegeneration via Activation of RhoA Pathway. PLoS ONE 8(1): e54692 https://doi.org/10.1371/journal.pone.0054692 23365678 10.1371/journal.pone.0054692 PMC3554698

